# Gender Matters: A State-of-the-Art Review of Transcatheter Structural Interventions in Older Women Vs Men

**DOI:** 10.1016/j.jacadv.2025.102330

**Published:** 2025-12-17

**Authors:** Jing Liu, Radmila Lyubarova, Jennifer Riggs, Jina Chung, Puja Kiran Mehta, Simrat Kaur, Abdulla Al Damluji, Vasilis Babaliaros, Leslie Cho

**Affiliations:** a Section of Cardiology, Department of Medicine, Michael E Debakey VA Medical Center, Baylor College of Medicine, Houston, Texas, USA; b Section of Cardiology, Department of Medicine, Albany Medical Health System, Albany, New York, USA; c Section of Cardiology, Minneapolis Heart Institute-St. Paul, St. Paul, Minnesota, USA; d Section of Cardiology, Harbor UCLA Medical Center, Torrence, California; e Section of Cardiology, Emory University, Atlanta, Georgia, USA; f Section of Cardiology, Cleveland Clinic, Cleveland, Ohio, USA

**Keywords:** aging, cardiovascular outcomes, frailty, sex differences, transcatheter structural heart interventions

## Abstract

With rising life expectancy, the population of older adults over the age of 65 is rapidly expanding, with women comprising the majority. Structural heart disease is common in this group, and transcatheter interventions have transformed its management. Optimal outcomes require careful consideration of sex-specific differences. This review examines transcatheter structural heart interventions in older adults with a focus on sex-based outcomes, procedural planning, and current knowledge gaps.

## Highlights

• Transcatheter structural interventions are safe and effective therapies for older patients ≥65 years of age.
• Sex-specific differences influence clinical presentation, procedural planning, and outcomes in transcatheter interventions.
• Comprehensive assessment of physical and cognitive functions preprocedure is needed to optimize cardiovascular outcomes.
• Older adults, especially women, remain underrepresented in clinical trials, necessitating future population level research.

With increasing life expectancy, most countries are experiencing growth of the older population.^1^ In the United States, adults ≥80 years represent the fastest-growing age group. Women comprise of majority of the older adults, exceeding 70% in some countries like Germany. Structural heart diseases such as severe aortic stenosis and valvular regurgitation affect approximately 10% of the population aged ≥75 years.^2^ Transcatheter interventions have revolutionized management of structural heart diseases in older adults. This review focuses on transcatheter interventions in older patients ≥65 years, exploring sex-specific differences in procedural planning, outcomes, and knowledge gaps (Central Illustration).

**Central Illustration:**
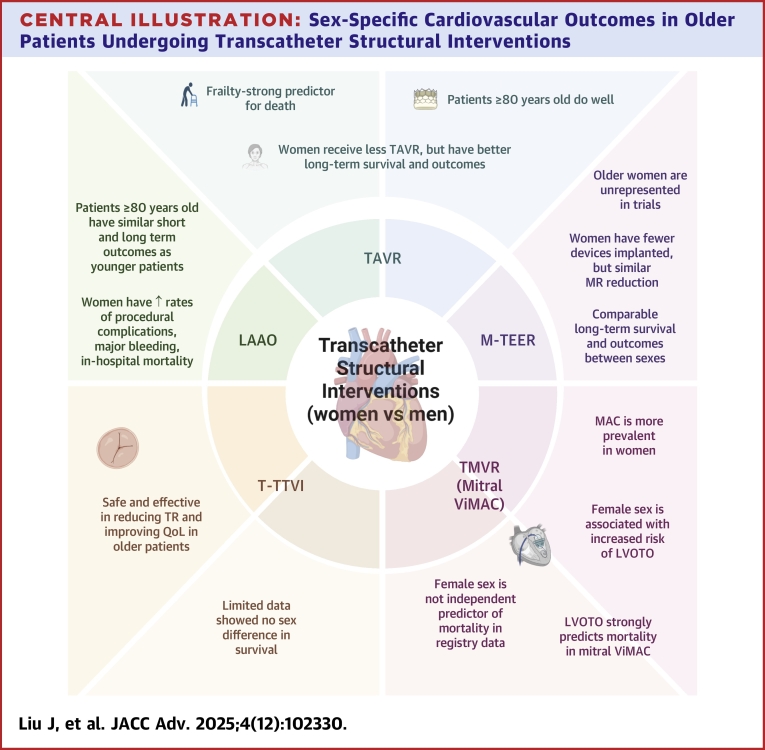
**Sex-Specific Cardiovascular Outcomes in Older Patients Undergoing Transcatheter Structural Interventions** LAAO = left atrial appendage closure; LVOTO = left ventricular outflow tract obstruction; Mitral ViMAC = mitral valve-in-mitral annular calcification; MR = mitral regurgitation; M-TEER = mitral transcatheter edge-to-edge repair; TMVR = transcatheter mitral valve replacement; TAVR = transcatheter aortic valve replacement; TR = tricuspid regurgitation; T-TTVI = transcatheter tricuspid valve interventions; MAC = mitral annular calcification.

## Aortic valve interventions

### Sex differences in clinical presentation and baseline comorbidities

In patients ≥75 years, approximately 12% have some degree of aortic stenosis (AS), and 3.4% have severe AS.^3^ Real-world data suggest transcatheter aortic valve replacement (TAVR) patients have a median age between 81 and 84 years of age.^4^ Majority of AS are calcific or degenerative, a minority is attributed to rheumatic or bicuspid processes. In older patients, classic AS symptoms—angina, syncope, and heart failure—might be obscured, and 25% of severe AS are asymptomatic.^5^ Sex differences are notable: women present at later stages with more symptoms, partly due to older age and different pathophysiologic responses to chronic pressure overload.

Women have smaller body surface area, worse renal function, and exhibit higher operative risk (Society of Thoracic Surgeons [STS] scores)^6^, ^7^, ^8^, ^9^ but have lower rates of diabetes, previous coronary artery bypass surgery, myocardial infarction, and atrial fibrillation compared to men.^10^ Women are also more frail, which has been found to be the strongest predictor of death, 1-year disability, and an independent predictor of major bleeding and transfusion following TAVR.^11^ Sedentary patients are often older, female, frailer, cognitively impaired, depressed, and have multiple comorbidities. Sarcopenia, which impacts recovery, as muscle strength is associated with shorter hospital stay and better 1 year health-related quality of life, is more common in men. Therefore, it is important to take into consideration the complete physical health, functional, neurocognitive, and socioenvironmental status in older men and women undergoing TAVR in order to systematically risk stratify and identify opportunities for optimization.^12^

### Imaging assessment

Echocardiographic assessment of AS is uniquely challenging in older women. Older women develop more concentric remodeling, characterized by increased wall thickness and reduced chamber size, resulting in smaller stroke volume and hence higher prevalence of paradoxical low-flow low-gradient AS. This can result in underdiagnosis and delay in treatment.^13^ Computed tomography (CT) provides accurate assessment of the extent and pattern of aortic valve calcification. However, the degree of aortic valve (AV) calcium to cause any degree of AS severity is significantly lower in women than in men likely because women have more aortic valve fibrosis.^14^^,^^15^ As a result, the severity of aortic stenosis in older women might be underestimated due to lower AV calcium on CT. Women also have more ascending aorta calcification and higher prevalence of porcelain aorta.^16^

### Procedural considerations

Multiple large-scale studies in the United States and Europe have demonstrated that women with severe AS are less likely to receive AVR (including TAVR) compared to men even after adjusting for age, comorbidities, and clinical presentation, likely reflecting underdiagnosis, delayed referral, and possible underestimation of disease severity in women.^8^^,^^17^

Historically, while some women had fallen out of the range of available devices due to smaller aortic valve area and annulus, this has been rectified by the introduction of smaller sizes across self and balloon-expandable valves. Studies have suggested superiority of TAVR in older women with smaller annulus compared to surgical aortic valve replacement (SAVR). The RHEIA (Randomized Research in Women All Comers with Aortic Stenosis) trial, first randomized controlled trial of TAVR in women, found balloon-expandable TAVR superior to SAVR for 1-year primary composite endpoint of all-cause death, stroke, or rehospitalization, but higher permeant pacemaker implantation rate.^18^ Hemodynamic performance and vascular complications were comparable to SAVR, though surgical root enlargement was not performed in SAVR.^19^ A pooled analysis of women enrolled in RHEIA and PARTNER (PARTNER 3 Trial: Safety and Effectiveness of the SAPIEN 3 Transcatheter Heart Valve in Low Risk Patients With Aortic Stenosis) 3 corroborated these findings, showing 1-year superiority of TAVR for the composite endpoint, again driven by lower rehospitalization, across a range of annular sizes and ages.^20^

Smaller annulus, friable tissue, and subaortic calcium increase the risk of aortic root rupture in older women, especially with balloon-expandable valves. Self-expanding devices are thus favored in women with smaller annuli and calcified iliofemoral vessels but associated with higher pacemaker rates and longer hospital stay.^21^ The SMART (Small Annuli Randomized To Evolut or Sapien) trial found self-expanding valves noninferior to balloon-expandable ones for 12-month outcomes of death, stroke, and heart failure hospitalization, with better hemodynamics and lower valve gradients.^22^ However, women’s smaller sinus of Valsalva dimension and lower coronary heights increase the risk of coronary obstruction. Baseline and procedural variables associated with coronary obstruction were older age, female sex, no previous coronary artery bypass graft, the use of a balloon-expandable valve, and previous surgical aortic bioprosthesis. Left coronary artery was the most involved.^23^

Women also have higher rates of vascular complications after TAVR due to smaller, more tortuous femoral arteries, and higher sheath-to-artery ratios.^15^^,^^24^ However, limited data exist on the benefits of nontransfemoral access in women.

### Short- and long-term outcomes

Women have worse short-term outcomes after TAVR, likely attributed to less favorable anatomy such as smaller vessel size leading to more vascular complications, along with higher prevalence of frailty and older age at the time of procedure. In a multiyear propensity-matched nationwide study, women had higher in-hospital mortality, stroke, postprocedural bleeding, vascular complications, pericardial complications, acute respiratory failure, need for transfusion, need for vasopressors, and major adverse cardiac and cerebrovascular events. These data are supported by other studies that similarly found women to have higher in-hospital mortality and major cardiovascular events, largely driven by vascular complications.^7^^,^^25^, ^26^, ^27^ Women also have higher 30-day readmission rate. However, pacemaker requirement was lower in women,^28^^,^^29^ likely due to women having shorter His-ventricular interval (HV) intervals and less frequent HV prolongation after TAVR.^30^^,^^31^ Despite worse short-term outcomes after TAVR, women have long-term survival advantage.^32^ In the SWEDEHEART (Swedish Web-system for Enhancement and Development of Evidence-based Care in Heart Disease Evaluated According to Recommended Therapies) study, the cumulative incidence of mortality at 1, 5, and 10 years was consistently lower in women.^33^ These findings are corroborated by other studies.^34^^,^^35^ Even among nonagenarians, those undergoing TAVR are more likely to be women and women do well with low in-hospital and 1-year mortality rates.^36^ Women’s more favorable long-term outcomes despite their worse short-term outcomes following TAVR are likely attributed to several factors: 1) lower baseline prevalence of comorbidities such as coronary artery disease, atrial fibrillation, and diabetes; 2) more favorable reverse remodeling with greater, earlier LV mass regression post-TAVR;^37^ and 3) lower incidence of moderate or severe paravalvular regurgitation, intervention, and improved prosthesis hemodynamics due to their smaller annular size, which reduces the chance of prosthesis undersizing compared to men.

Following TAVR, most older patients suffer functional decline despite replacement of the aortic valve, with worsening frailty, sarcopenia, and disability.^38^, ^39^, ^40^ Cardiac rehabilitation, generally well-tolerated in older patients, is crucial in improving the functional status and quality of life. Despite strong evidence, post-TAVR cardiac rehabilitation referral remains low compared to surgical valve replacement. Women are more likely to have physical but not cognitive or psychosocial frailty and are independently more likely to be discharged to rehabilitation facilities. Despite this, women’s functional status was similar to men at 12 months.^41^

In summary, women with severe AS often present later with more symptoms and face anatomical and physiological challenges. Despite better long-term outcomes, they receive TAVR less often—possibly due to underdiagnosis. Moving forward, sex-specific diagnostic criteria, tailored risk stratification, and inclusive trial design are essential to optimize outcomes for women undergoing TAVR.

## Mitral Valve Interventions

Mitral regurgitation (MR), the most common valve disease in older adults affecting 1 to 2% of the global population, has a predilection of women.^42^^,^^43^ Women present with more complex mitral valve pathology, including increased leaflet thickening and anterior or bi-leaflet prolapse. Mitral annular calcification (MAC) is also more prevalent in older women, possibly due to metabolic and hormonal changes, and often present as mixed mitral valve disease.^44^^,^^45^

### Mitral transcatheter edge-to-edge repair

Mitral transcatheter edge-to-edge repair (M-TEER) has emerged as a safe and effective therapy for patients with severe primary MR at high or prohibitive surgical risk, and in those with symptomatic ≥ moderate-to-severe MR despite optimal medical therapy.^46^^,^^47^ The aforementioned anatomical challenges may have contributed to the underrepresentation of women in key M-TEER clinical trials such as COAPT (Cardiovascular Outcomes Assessment of the MitraClip Percutaneous Therapy) and EVEREST II (Pivotal Study of a Percutaneous Mitral Valve Repair System [EVERESTIIRCT]) (with 36% and 38% women representation), even though population data showed 52% of severe MR population are women.^48^ The landmark studies on M-TEER did not find sex-based difference in outcomes but were underpowered to do so.

In registry data, women who underwent M-TEER were found to be older, had fewer comorbidities, higher baseline ejection fraction, lower functional status, and more baseline NYHA functional class III/IV symptoms than men.^49^^,^^50^ Anatomic challenges in women include smaller mitral annular dimension, more frequent mitral stenosis or mixed pathology, which may limit the number of clips that can be safely implanted and increase the risk of elevated postprocedural gradients.^51^ However, women still achieve similar degree of MR reduction compared to men.^50^^,^^52^ Some studies report women having higher rates of periprocedural bleeding^52^^,^^53^ and 90-day readmission rate compared to men.^54^ However, meta-analysis has shown no significant sex differences in 30-day mortality, unadjusted mortality, and heart failure hospitalization at 12 months; women had lower incidence of adjusted mortality.^49^ The American College of Cardiology (ACC)/STS TVT (Society of Thoracic Surgeons Transcatheter Valve Therapy) registry also found women had lower long-term adjusted 1-year all-cause mortality, and similar rate of major adverse cardiovascular events compared to men.^50^ In summary, despite baseline differences and anatomical challenges, women undergoing M-TEER women have similar 30-day mortality, and similar or better long-term survival compared to men.

### Transcatheter mitral valve replacement

Transcatheter mitral valve replacement (TMVR) is an emerging therapy for high-risk patients with symptomatic severe mitral valve disease (MR, mitral stenosis, or mixed) due to severe MAC (Valve-in-Mitral Annular Calcification [ViMAC]), who are deemed high surgical risk and have unfavorable anatomy for M-TEER. Compared to those with failed annuloplasty rings (Mitral Valve-in-Ring) or failed bioprosthetic mitral valves (Mitral Valve-in-Valve), ViMAC is associated with lower procedural success and higher in-hospital and 30-day mortality.^55^ This has significant clinical impact, especially for older women, in whom MAC is more prevalent.^56^

The earliest and most comprehensive outcome data for mitral ViMAC come from the TMVR in MAC Global Registry (68% female). All-cause mortality at 30 days and 1 year were 25% and 53.7%. Among participants selected for the trial, female sex was not an independent predictor of 1-year mortality. However, left ventricular outflow tract (LVOT) obstruction (LVOTO) is an Achilles heel of ViMAC and found to be a strong independent predictor for 30-day and 1-year mortality in the TMVR in MAC Global Registry.^57^ In a subanalysis of the registry, female sex was found to be strongly associated with increased risk of LVOTO.^58^ Additionally, risk of LVOTO is also a primary reason for screen failure, with one study noting 67% of screened failed patients were women.^59^ Procedural techniques to preemptively mitigate the risk of LVOTO during TMVR, such as alcohol septal ablation before TMVR, anterior leaflet laceration during TMVR (LAMPOON technique), anterior leaflet resection or septal myectomy during TMVR, and radiofrequency ablation of the basal ventricular septum could potentially expand eligibility and improve outcomes for women undergoing ViMAC.^58^

Unfortunately, data on sex-specific outcomes in ViMAC remain limited. The STS/ACC TVT and TMVR registries reported no sex difference in overall TMVR mortality, but sex-specific outcomes for ViMAC were not analyzed due to the limited number of participants.^55^^,^^60^ Future prospective studies adequately powered to study gender-specific outcomes are warranted.

## Tricuspid valve interventions

Despite having smaller cardiac chamber sizes, women are more likely to develop tricuspid regurgitation (TR), possibly due to hormonal influences with aging, particularly estrogen loss, affecting extracellular matrix remodeling and right heart adaptation.^61^ Women are older at presentation and have worse functional status.^62^ While some studies show higher mortality in men with TR, others have found no sex-based differences.^63^^,^^64^

Surgical tricuspid valve annuloplasty remains the standard of care for severe functional TR, but many older patients are ineligible due to high operative risk. Innovations in catheter-based techniques (transcatheter tricuspid valve intervention [TTVI]) have emerged as alternatives, including tricuspid transcatheter edge-to-edge repair (T-TEER) and transcatheter tricuspid valve replacement. The TRILUMINATE Pivotal trial (mean age 78 years, 58.9% women) showed that T-TEER with Triclip significantly reduced TR and improved functional status and quality of life (QoL) at 1 year.^65^ Similarly, in a European T-TEER registry (mean age 79 years, 53% women) treatment of severe TR using the PASCAL system showed sustained TR reduction and improved 1-year clinical status.^66^ In TRISCEND (Edwards EVOQUE Transcatheter Tricuspid Valve Replacement: Pivotal Clinical Investigation of Safety and Clinical Efficacy using a Novel Device) study (mean age 79.3 years, 76.8% female), transfemoral EVOQUE TV replacement resulted in reduced TR, increased stroke volume and cardiac output, improved survival, functional, and QoL outcomes at 1 year,^67^ with TR reduced to ≤ mild in almost all of the participants (97.6%). In TRISCEND II trial (mean age 79.2 years, 75.5% women), transcatheter tricuspid valve replacement was superior to medical therapy for symptom improvement and QoL at 1 year, though procedural complications such as bleeding and new pacemaker implantation were more frequent.^68^ A study of 702 patients (386 women) undergoing TTVI showed similar survival by sex, but differences in prognostic markers. Women with a tricuspid annular plane systolic excursion/mean pulmonary arterial pressure ratio <0.612 had higher 2-year mortality risk, compared to men with a threshold <0.434.^69^ Further studies are needed to better understand sex-specific mechanisms and prognostic thresholds in transcatheter interventions for TR, particularly as women remain the majority of TTVI recipients.

## Left atrial appendage occlusion

Left atrial appendage occlusion (LAAO) has emerged as a viable alternative to lifelong anticoagulation in patients with atrial fibrillation.^70^ Currently, a third of patients undergoing LAAO implantation are ≥80 years of age.^71^ Anatomical differences between men and women can make the procedure more technically challenging for women, particularly older women. Women have thinner, more fragile left atrial and left atrial appendage walls, with smaller orifices and a more tubular shape.^72^ They also typically have smaller vessel diameters, making access more difficult.^73^ Women with AF also experience more severe symptoms and have higher stroke rates.^74^

Recent data from 2 major trials (PROTECT-AF [Percutaneous Closure of the Left Atrial Appendage Versus Warfarin Therapy for Atrial Fibrillation], PREVAIL [Prospective randomized evaluation of the Watchman Left Atrial Appendage Closure device in patients with atrial fibrillation versus long-term warfarin therapy: the PREVAIL trial]) and registry data have shown similar short- and long-term outcomes for patients over and below 80 years of age.^75^ However, important sex differences in outcomes exist. Women are older with worse risk profiles and higher rates of prior stroke.^76^ From the PROTECT AF trial, a sex-specific subgroup analysis showed significant reduction in the composite endpoint for men, but no such difference in women.^77^ Women tend to have longer hospital stays, increased in-hospital mortality, and increased major adverse events, such as major bleeding; pericardial effusion requiring drainage are twice as common as in men.^76^ Analysis of the Nationwide Readmission Database further revealed that women experience higher rates of in-hospital death, major vascular complications, and pericardial complications.^78^ Future research should focus on optimizing LAAO outcomes in older women, addressing anatomical and procedural challenges that contribute to their higher complication rates and poorer postprocedural outcomes compared to men.

## Conclusions

Transcatheter structural heart interventions are generally safe and effective in older adults; however, important sex-based differences in outcomes persist. Women often face underdiagnosis and delayed referral, which may contribute to worse short-term outcomes for some procedures. Despite this, studies have shown that women can achieve comparable—or even superior—long-term outcomes. Bridging these gaps will require targeted, population-based research to support more personalized and equitable care.

Perspectives **COMPETENCY IN PATIENT CARE AND PROCEDURAL SKILLS:** Clinicians caring for older adults undergoing transcatheter structural interventions should integrate sex-specific anatomical, physiological, and age-appropriate risk profiles into procedural planning and decision-making. Competency includes recognizing that older women can present with distinct clinical and risk profiles. Providers should tailor imaging, access strategy, valve selection, and postprocedural care accordingly. Competent practice also requires awareness of sex-based disparities in referral, trial representation, and outcomes to ensure equitable, evidence-based treatment for both women and men.

**TRANSLATIONAL OUTLOOK:** Future research should prioritize prospective studies powered to detect sex-specific differences in structural heart interventions. Development of sex-specific diagnostic thresholds, imaging criteria, and risk stratification tools is needed to improve quality of care in older women, who remain susceptible to underdiagnosis and delayed referral. Advances in device design and procedural techniques should also incorporate sex-related anatomical considerations to reduce complications. Broader inclusion of women—particularly the oldest-old—in clinical trials will be essential to refine therapeutic strategies and optimize outcomes for the growing population of older adults with structural heart disease.

**Table 1:** Sex-Based Procedural Considerations and Outcomes in Transcatheter Structural Interventions

Intervention	Key Sex-Based Anatomical Differences	Procedural Considerations	Short-Term Outcomes	Long-Term Outcomes
TAVR (transfemoral aortic valve replacement)	Women: • Smaller annulus • Smaller vessels • Smaller sinus of Valsalva, more concentric LV remodeling • Lower AV calcium, higher prevalence of porcelain aorta • More frail	• Self-expanding valves favored in women with small annuli • Detailed imaging to reduce vascular complications • Higher risk of coronary obstruction	Women: • Higher in-hospital mortality, vascular complications, bleeding, 30-day readmission • Lower pacemaker rates	• Better long-term survival in women
M-TEER (mitral transcatheter edge-to-edge repair)	Women: • More anterior/bileaflet prolapse • More MAC	Women: • Fewer clips used due to higher gradient • Shorter procedure time	Women: • More periprocedural bleeding, 90-day readmission • Similar short-term mortality as men	• Comparable or better mortality • Similar adverse cardiovascular events
TMVR (transcatheter mitral valve replacement)	Women: • More MAC • Smaller LV/LVOT	• Higher risk of LVOT obstruction • May require anterior leaflet laceration, septal reduction	• Limited data • Female sex not a predictor of mortality in ViMAC • Female sex not a predictor of mortality in TMVR	
TTVI (transcatheter tricuspid valve intervention)	Women: • More likely to develop TR • More frail • Worse functional status	• More women undergo TTVI • Most achieve TR reduction to ≤ mild	• Similar survival by sex	• Increased cardiac output, improved survival, and QoL outcomes at 1 year
LAAO (left atrial appendage occlusion)	Women: • Smaller, more fragile LA and LAA • Smaller orifice • More tubular shape-LAA	• Procedure may be more technically challenged in women	Women: • Higher in-hospital mortality, vascular and pericardial complications	• No sex difference in stroke and CV death

AV = aortic valve; CV = cardiovascular; LA = left atrium; LAA = left atrial appendage; LV = left ventricle; LVOT = left ventricular outflow tract; MAC = mitral annular calcification; TR = tricuspid regurgitation; ViMAC = Valve-in-Mitral Annular Calcification.

## Funding support and author disclosures

Dr Damluji has received research funding from the Pepper Scholars Program of the Johns Hopkins University Claude D. Pepper Older Americans Independence Center (OAIC) funded by the 10.13039/100000049NIA P30-AG021334; and has received mentored patient-oriented research career development award from the 10.13039/100000050National Heart, Lung, and Blood Institute K23-HL153771-01. All other authors have reported that they have no relationships relevant to the contents of this paper to disclose.
